# Early versus adult onset of schizophrenia: an examination of premorbid and current IQ

**DOI:** 10.1016/j.scog.2025.100397

**Published:** 2025-10-14

**Authors:** Tereza Calkova, Anja Vaskinn, Lynn Mørch-Johnsen, Runar Elle Smelror, Kjetil Nordbø Jørgensen, Laura A. Wortinger, Simon Cervenka, Karin Collste, Beathe Haatveit, Christine Mohn, Anne Margrethe Myhre, Erik G. Jönsson, Nils Eiel Steen, Ole A. Andreassen, Ingrid Melle, Ingrid Agartz, Torill Ueland, Dimitrios Andreou

**Affiliations:** a Centre for Psychiatry Research, Department of Clinical Neuroscience, Karolinska Institutet & Stockholm Health Care Services, Region Stockholm, Stockholm, Sweden; b Region Vastmanland – Uppsala University, Centre for Clinical Research, Vastmanland Hospital Vasteras, Västerås, Sweden; c Norwegian Centre for Mental Disorders Research (NORMENT), Institute of Clinical Medicine, University of Oslo, Oslo, Norway; d Centre for Precision Psychiatry, Institute of Clinical Medicine, University of Oslo, Norway; e Centre for Research and Education in Forensic Psychiatry, Oslo University Hospital, Oslo, Norway; f Department of Psychiatry & Department of Clinical Research, Østfold Hospital, Grålum, Norway; g Division of Mental Health and Addiction, Vestre Viken Hospital Trust, Drammen, Norway; h Department of Psychiatric Research, Diakonhjemmet Hospital, Oslo, Norway; i Department of Medical Sciences, Psychiatry, Uppsala University, Uppsala, Sweden; j Section for Clinical Psychosis Research, Division of Mental Health and Addiction, Oslo University Hospital, Oslo, Norway; k National Centre for Suicide Research and Prevention, Institute of Clinical Medicine, University of Oslo, Norway; l Departement of Research and innovation, Division of Mental Health and Addiction, Oslo University Hospital, Oslo, Norway; m Child and Adolescent Psychiatry Unit, Institute of Clinical Medicine, University of Oslo, Oslo, Norway; n Department of Psychology, University of Oslo, Norway

**Keywords:** Early onset schizophrenia, Adult onset schizophrenia, IQ, Cognition

## Abstract

**Background:**

Cognitive deficits are core findings in schizophrenia, but whether the severity of impairments is related to the age of onset remains unclear. We hypothesized that early onset schizophrenia (EOS; onset before age 19) is associated with lower IQ compared to adult-onset schizophrenia (AOS; onset from age 19).

**Methods:**

We included 99 adult patients with EOS (age of onset: 15.3 ± 2.8 years), 282 adult patients with AOS (age of onset: 26.5 ± 7.4 years), and 863 adult healthy controls (HC). We assessed current IQ with Wechsler Abbreviated Scale of Intelligence (WASI) and estimated premorbid IQ with National Adult Reading Test (NART).

**Results:**

Both patient groups had lower current IQ than HC (*p* < 0.001). Full-scale (*p* = 0.004), performance (*p* = 0.003) and verbal (*p* = 0.011) current IQ were significantly lower in EOS than in AOS, with 5 IQ units difference for all three measures. EOS and AOS did not differ in premorbid IQ, but EOS showed a steeper IQ decline from premorbid levels than AOS (11.4 vs. 8 IQ units, respectively, *p* = 0.013).

**Conclusion:**

EOS had lower current IQ than AOS, but did not differ in premorbid IQ, suggesting a larger decline from premorbid IQ levels. This could imply different neurodevelopmental processes underlying cognitive dysfunction related to age of onset in schizophrenia, underscoring the necessity for further inquiry into the mechanisms driving this decline and strategies for its prevention.

## Highlights

• Early-onset schizophrenia (EOS) is associated with lower IQ than adult-onset (AOS).
• EOS has a steeper decline in IQ from premorbid levels compared to AOS.
• study suggests relations of cognitive dysfunction to age of onset in schizophrenia.

## 1 Introduction

Schizophrenia is a severe psychiatric disorder with increasing global burden affecting approximately 0.3 % of the human population and usually debuting in young adulthood (Charlson et al., 2018). However, a notable subset of patients experience their initial psychotic episode before reaching adulthood. The disorder is then termed early-onset schizophrenia (EOS)(Werry et al., 1991). EOS is recognized as a potentially more severe form with a less favourable prognosis compared to adult-onset schizophrenia (AOS) (Clemmensen et al., 2012; Diaz-Caneja et al., 2015). For instance, EOS has been associated with more prominent negative symptoms and a higher rate of premorbid impairments (Ballageer et al., 2005; Hollis, 2003; Schimmelmann et al., 2007).

Although not part of the Diagnostic and Statistical Manual of Mental Disorders (DSM) criteria, cognitive deficits are considered core features of schizophrenia (Green et al., 2019; Kahn et al., 2015). Cognitive impairments are evident premorbidly (Khandaker et al., 2011; Mollon and Reichenberg, 2018; Woodberry et al., 2008) and are closely related to the functional impairments associated with the disorder (Bowie and Harvey, 2006). At the same time, schizophrenia is characterized by cognitive heterogeneity, with variability seen from the first episode stage (Mesholam-Gately et al., 2009), even before initiation of antipsychotic treatment (Lee et al., 2024). Several studies have defined patient subgroups characterized by IQ (intelligence quotient) differences. Patients with schizophrenia exhibit a spectrum of IQ scores ranging from preserved to severely impaired (Ayesa-Arriola et al., 2018; Carruthers et al., 2019). Moreover, beyond disparities in current IQ levels, it has also been shown that patients with schizophrenia can also differ in their trajectories of cognitive functioning over time. A recent longitudinal study, with a partly overlapping sample with the present study, reported lower premorbid IQ (assessed retrospectively) relative to healthy controls (HC), a sharp IQ decline from premorbid levels to baseline assessment, and an IQ increase at 10-year follow-up (Flaaten et al., 2022). However, this study did not consider subgroups, and there are in fact several studies that have suggested the existence of three distinct patient groups categorized based on IQ levels and its course (Ohi et al., 2017; Vaskinn et al., 2020; Weickert et al., 2000; Wells et al., 2015). According to this research, one group is characterized by a deterioration of IQ from premorbid levels, another group shows preserved IQ, and a third group, has low scores both premorbidly and after illness onset. Interestingly, some studies have found continued deterioration of cognitive abilities after the onset of psychosis (Fujino et al., 2017; Kremen et al., 2010; Meier et al., 2014), while others do not find evidence for further deterioration (Bora and Murray, 2014; Caspi et al., 2003).

Differences in the magnitude and trajectories of cognitive impairment illustrate the heterogeneity of cognitive ability in schizophrenia. In this context, an adolescent contra an adult age of disease onset may be one underlying factor. The early timing of illness onset may disrupt brain development and aggravate cognitive deficits considering that adolescence is a period of abundant neurodevelopment (Arain et al., 2013), and that cognitive aberrations in schizophrenia may be related to neurodevelopmental disturbances (Melle, 2019).

Previous reports have explored cognition in relation to age of onset, with mixed findings. According to a meta-analysis, patients with EOS have lower IQ and larger impairments in arithmetic and executive functions, speed of processing and verbal memory, compared to AOS patients (Rajji et al., 2009). However, there are also studies that report no significant cognitive differences between EOS and AOS patients (Coulon et al., 2020; Holmen et al., 2012). The time-point when the EOS patients are assessed, i.e., during adolescence close to illness-onset versus later in adulthood, may be one reason for such discrepancies. It could be that the EOS patients´ cognition is still developing after the onset of psychosis and is not easily compared with already mature adult patients.

In this study, we examined current and premorbid intellectual functioning in adult patients with either EOS or with AOS, and in adult healthy controls (HC). In addition, premorbid cognitive functioning was assessed retrospectively using a reading test. We hypothesized that patients with EOS would have lower IQ scores than AOS patients. Further, we anticipated patients to show decline from premorbid to current IQ levels, with a steeper decline in EOS that could reflect more severe alterations in neurodevelopment.

## 2 Methods

### 2.1 Participants

We included 381 adult patients with schizophrenia spectrum disorders, mean age 30.4 years (9.9) and 863 HC, mean age 33.3 years (9.2). Among patients, 99 were defined as EOS (age of onset of psychosis before age 19) mean age of onset 15.26 years (2.8) and 282 as AOS (age of onset of psychosis from age 19) mean age of onset 26.52 years (7.4). In terms of specific diagnoses, 286 patients had schizophrenia, 35 schizophreniform disorder and 60 schizoaffective disorder. Patients were recruited from outpatient and inpatient psychiatric units in Oslo, Norway, as part of the Thematically Organised Psychosis (TOP) research study previously part of the Norwegian Centre for Mental Disorder Research (NORMENT). HC were recruited from the same catchment area using the national population register of Norway.

The following exclusion criteria were applied for all participants: previous moderate or severe head injury, neurological disorders or medical conditions that could affect brain function, and mental disability (defined as full-scale IQ < 70). Further, only participants with Norwegian as native language or those who had completed all their formal education in Norway were included. Participants with dyslexia were excluded. HC with alcohol or drug abuse, history of severe mental illness, or having relatives with severe mental illness were also excluded. The Primary Care Evaluation of Mental Disorders (Prime-MD) (Spitzer et al., 1994) was used for screening HC.

### 2.2 Clinical and cognitive measures

Clinical assessments were conducted by trained medical doctors and psychologists. The Structured Clinical Interview for DSM-IV axis I disorder (SCID-I) module A–E (Spitzer et al., 1989) was used for diagnostic assessment. Age of disease onset, the duration of illness and the duration of untreated psychosis were obtained from the clinical interviews. Data were also obtained on current antipsychotic medication use. For patients on antipsychotics the current chlorpromazine equivalent doses (CPZ) were calculated (Andreasen et al., 2010). Further, alcohol use was evaluated with the Alcohol Use Disorders Identification Test (AUDIT) (Bohn et al., 1995), drug use with the Drug Use Disorder Identification Test (DUDIT) (Berman et al., 2005) and psychotic symptoms with the Positive and Negative Syndrome Scale (PANSS) (Kay et al., 1987).

IQ was assessed by clinical psychologists or trained research personnel with formal training in standardized neuropsychological testing. Current IQ was measured with the four-subtest Wechsler Abbreviated Scale of Intelligence (WASI) (Wechsler, 2007), yielding scores for full scale IQ (FIQ), performance IQ (PIQ) and verbal IQ (VIQ). Premorbid IQ was estimated with the National Adult Reading Test (NART) (Sundet and Vaskinn, 2008). IQ difference was calculated as NART IQ minus current WASI FIQ.

### 2.3 Statistics

Differences between EOS and AOS in clinical and demographic characteristics (sex, age, AUDIT and DUDIT scores, duration of illness, duration of untreated psychosis, PANSS total score and medication variables) were examined with chi-square tests (categorical variables) and *t*-tests (continuous variables). The correlations between each of these variables and the WASI scores in the two clinical samples collapsed were examined with point-biserial (r_pb_) (categorical variables) and Spearman's (r_s_) (continuous variables) correlation analyses.

Our first research aim concerned differences between EOS and AOS in current IQ. This was examined in two steps. First, a series of preliminary analyses of variance (ANOVAs) compared the three groups (EOS, AOS, HC) for the three current IQ measures (WASI FIQ, WASI PIQ, WASI VIQ). Diagnostic group was the independent variable, and the WASI IQ scores were entered as the dependent variable. In the second step, we applied analyses of covariance (ANCOVAs), controlling for variables that were significantly correlated with IQ in the initial correlational analyses. Diagnostic group was again the independent variable but consisted of only EOS and AOS this time. As IQ measures are already determined based on age-specific norms, age was not inserted as a covariate. The second step involved three ANCOVAs (WASI FIQ, WASI PIQ and WASI VIQ). We therefore accepted statistical significance at a Bonferroni-adjusted alpha level of 0.017 (0.05/3).

Our second research aim was to investigate whether EOS and AOS differed in premorbid IQ and in estimated IQ decline. We here applied t-tests and assessed the main effect of diagnostic status (EOS vs. AOS) on the estimated premorbid IQ and IQ decline.

Effect sizes (partial eta-squared; η^2^) are reported. We conducted all the analyses with IBM SPSS Statistics 28.

## 3 Results

Demographics and clinical characteristics of patients and HC are presented in Table 1, Table 2. On average, patients were younger than HC (Table 1), while patients with EOS were younger, had higher AUDIT score, longer duration of illness, longer duration of untreated psychosis and higher PANSS total score compared with patients with AOS (Table 2).

**Table 1:** Demographics and clinical characteristics of adult patients with schizophrenia spectrum disorders and healthy controls. Group differences in sex distribution and age between patients and healthy controls are shown. For patients, age of onset, duration of illness (DOI), duration of untreated psychosis (DUP), the percentage of patients on antipsychotics as well as the chlorpromazine equivalent doses (CPZ), and Positive and Negative Syndrome Scale (PANSS) total scores are presented. *P* values <0.05 shown in bold.

	Patients		Healthy controls		P-value^b^
	N^a^	Mean (SD) or %	N^a^	Mean (SD) or %	
Sex (% females)	381	46.2 %	863	47.9 %	0.588
Age (years)	381	30.4 (9.9)	863	33.3 (9.2)	**<0.001**
Age of onset (years)	381	21.9 (8.3)			
DOI (years)	381	6.7 (7.4)			
DUP (days)	245	189 (286)			
On antipsychotics (%)	381	88.7 %			
CPZ (mg/day)	376	321.7 (273.6)			
PANSS total score (SD)	376	63.2 (16.5)			

a Number of participants with data for each variable.

b Chi-square test or *t*-test.

**Table 2:** Group differences between adult patients with early-onset schizophrenia (EOS) and adult patients with adult-onset schizophrenia (AOS) in sex, age, alcohol use disorder identification test (AUDIT) score, drug use disorder identification test (DUDIT) score, duration of illness (DOI), duration of untreated psychosis (DUP), Positive and Negative Syndrome Scale (PANSS) total score, the percentage of patients on antipsychotics as well as the chlorpromazine equivalent doses (CPZ). Correlations of each variable with performance IQ (PIQ), verbal IQ (VIQ) and full-scale IQ (FIQ) are also shown. P values <0.05 shown in bold.

	EOS		AOS		P-value^b^	Correlation with PIQ		Correlation with VIQ		Correlation with FIQ	
	N^a^	Mean (SD) or %	N^a^	Mean (SD) or %			P-value^c^		P-value^c^		P-value^c^
Sex (% women)	99	52.5 %	282	44 %	0.142	−0.155	**0.002**	+0.008	0.879	−0.075	0.146
Age (years)	99	25 (8)	282	32.3 (9.7)	**<0.001**^d^	+0.048	0.354	+0.192	**<0.001**	+0.134	**0.009**
AUDIT	63	9.4 (8)	147	6.2 (6.3)	**0.006**^d^	−0.061	0.378	−0.132	0.056	−0.105	0.130
DUDIT	62	5.2 (10.2)	156	3.4 (6.3)	0.180^d^	+0.125	0.066	−0.052	0.443	+0.043	0.532
DOI (years)	99	9.6 (8.9)	282	5.7 (6.6)	**<0.001**^d^	−0.011	0.836	+0.147	**0.004**	+0.084	0.101
DUP (days)	73	292.2 (312.9)	172	145.2 (262.7)	**<0.001**^d^	−0.082	0.202	−0.023	0.724	−0.054	0.399
PANSS total score	98	66.1 (14.6)	278	62.2 (17.1)	**0.034**^d^	−0.096	0.063	−0.190	**<0.001**	−0.167	**0.001**
On antipsychotics (%)	99	86.9	282	89.4	0.500	−0.036	0.483	+0.013	0.799	+0.016	0.762
CPZ (mg/day)	98	308 (255.6)	278	326.5 (280)	0.566	−0.104	**0.043**	−0.047	0.368	−0.092	0.076

a Number of participants with data for each variable.

b Chi-square test or t-test.

c Point-biserial correlations for binary variables; Spearman's correlations for quantitative variables.

d Mann-Whitney *U* test was also run due to unequal variances, and confirmed the *t*-test result.

Age (r_s_ = 0.134, *p* = 0.009) was positively correlated with FIQ, while PANSS total score (r_s_ = −0.167, *p* = 0.001) was inversely correlated with FIQ. Sex was correlated with PIQ (r_pb_ = −0.155, *p* = 0.002) with men having higher PIQ than women. CPZ was inversely correlated with PIQ (r_s_ = −0.104, *p* = 0.043). Age (r_s_ = 0.192, *p* < 0.001) and DOI (r_s_ = 0.147, *p* = 0.004) were positively correlated with VIQ, while PANSS total score (r_s_ = −0.190, p < 0.001) was inversely correlated with VIQ (Table 2).

**Fig. 1:**
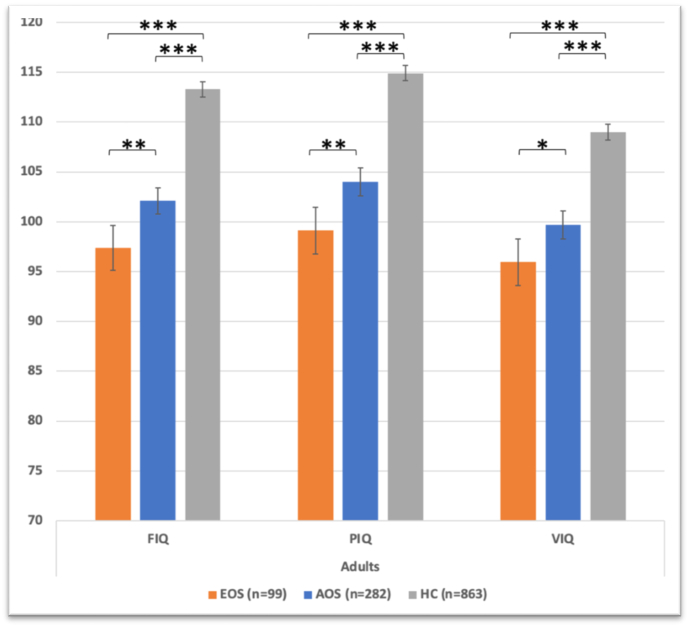
Full-scale IQ (FIQ), performance IQ (PIQ) and verbal IQ (VIQ) in adult patients with early-onset schizophrenia (EOS) and adult-onset schizophrenia (AOS), and adult healthy controls (HC). Estimated means with 95 % confidence intervals from analyses of variance are shown. There were two missing values for PIQ and VIQ among HC.

In the analyses of our first research aim, i.e., of differences in current IQ, the ANOVAs in the first step showed that both patient groups had significantly lower FIQ, PIQ and VIQ than HC (Table 3 & Fig. 1). In the second step, for WASI FIQ, the PANSS-adjusted ANCOVA showed a significant main effect of diagnostic group (F(1,373) = 8.204, p = 0.004, η^2^ = 0.022). For WASI PIQ, the sex- and CPZ-adjusted ANCOVA provided a significant main effect of diagnostic group (F(1,372) = 8.972, *p* = 0.003, η^2^ = 0.024). Finally, for WASI VIQ, the DOI- and PANSS-adjusted ANCOVA yielded a significant main effect of diagnostic group (F(1,372) = 6.548, *p* = 0.011, η^2^ = 0.017). For all the analyses in step 2, EOS had significantly lower current IQ than AOS. In ANCOVAs further adjusted for duration of illness and duration of untreated psychosis, which were both significantly longer in EOS compared to AOS (Table 2), EOS patients still showed significantly lower FIQ, PIQ, and VIQ (F(1,239) = 5.26, *p* = 0.023; F(1,236) = 4.20, *p* = 0.042; F(1,239) = 4.88, *p* = 0.028, respectively).

**Table 3:** Full-scale IQ (FIQ), performance IQ (PIQ) and verbal IQ (VIQ) in adult patients with early-onset schizophrenia (EOS), adult-onset schizophrenia (AOS) and adult healthy controls (HC). Estimated marginal means and standard errors from the analyses of covariance (ANCOVAs) are presented.

	EOS	AOS	HC	
N	99	282	863	
ANOVAs with pairwise comparisons				
FIQ	97.4 (1.1)	102.1 (0.7)	113.3 (0.4)	EOS < AOS<HC^b^
PIQ	99.1 (1.2)	104 (0.7)	114.9 (0.4)^a^	EOS < AOS<HC^b^
VIQ	96 (1.2)	99.7 (0.7)	109 (0.4)^a^	EOS < AOS<HC^c^

a Two missing values.

b *p* = 0.001 for EOS vs. AOS; *p* < 0.001 for EOS vs. HC and AOS vs. HC.

c p = 0.024 for EOS vs. AOS; p < 0.001 for EOS vs. HC and AOS vs. HC.

For our second research aim, we found that EOS and AOS did not differ in premorbid NART IQ, but EOS had a significantly larger IQ decline from premorbid to current levels compared to AOS (3.4 units larger IQ decline in EOS, *p* = 0.013) (Table 4).

**Table 4:** Estimated premorbid IQ and IQ decline in adult patients with early-onset schizophrenia (EOS) and patients with adult-onset schizophrenia (AOS). *P*-values of *t*-tests are demonstrated with values <0.05 shown in bold.

	EOS	AOS	P-value
N	99	282	
Premorbid IQ (SD)	108.8 (6.3)	110 (7.3)	0.109^a^
IQ decline (SD)	11.4 (11.7)	8 (12.1)	**0.013**

a Mann-Whitney *U* test was also run due to unequal variances, and confirmed the t-test result (*p* = 0.08).

## 4 Discussion

In the present study of patients with schizophrenia spectrum disorders, we found that adults with EOS had significantly lower current IQ than adults with AOS (Fig. 1). This was the case for FIQ, PIQ and VIQ, where the differences amounted to 5 IQ points for all measures. Whereas EOS and AOS did not differ in premorbid IQ, EOS showed a significantly larger decline from premorbid to current levels than AOS (11 and 8 IQ units, respectively).

Previous cross-sectional reports have assessed cognitive functioning in EOS vs. AOS with somewhat conflicting results, including those that have evaluated IQ. In a meta-analysis of studies assessing patients at their first episode of psychosis, patients with EOS showed larger deficits in various cognitive areas including IQ relative to patients with AOS (Rajji et al., 2009). One study, which included a smaller sample of the current AOS dataset, assessed patients with schizophrenia at their first treatment of psychosis and found no significant difference in current IQ between EOS and AOS (Holmen et al., 2012). Of studies that assessed cognition several years after illness onset, Basso and colleagues (Basso et al., 1997) found poorer cognitive performance, including lower current IQ, in EOS compared to AOS. Assessments took place 10 and 8 years after the onset of psychosis, respectively. Our study, which assessed adults with an illness duration of 9.6 years (8.9) and 5.7 years (6.6), for EOS and AOS, respectively, aligns with these results. Biswas and colleagues assessed patients with childhood-onset, adolescent-onset, and adult-onset schizophrenia 6 years after illness onset (Biswas et al., 2006). Childhood-onset patients (onset before age 14) showed significantly lower IQ than adolescent-onset schizophrenia patients (onset between 14 and 17 years of age) and AOS patients, while difference between IQ of adolescent-onset and AOS was not significant. In a recent study, Coulon and colleagues investigated IQ in EOS and AOS patients 10–12 years after illness onset (Coulon et al., 2020). In contrast to our result, they found no significant difference in IQ between the two groups.

The discrepancies in the literature may be due to when, in respect to illness onset, the IQ assessment is done. We do not know when a putative decline may occur. Also, sample differences may account for the mixed findings. For instance, previous studies are not in agreement about the cut-off used to assign a patient to early- or adult-onset psychosis groups. The applied cut-off has varied from 17 to 25 years of age, which may also have contributed to the inconsistent findings.

We did not identify differences in premorbid IQ between EOS and AOS. This indicates that premorbid cognitive heterogeneity is not likely to be explained by age at illness onset. It does not preclude, however, that age at illness onset is among the drivers of cognitive heterogeneity seen later in the illness course. Indeed, EOS had a larger decline from premorbid levels, although the AOS group also declined. This leads to the hypothesis that patients who experience an early onset of schizophrenia tend to exhibit a more rapid decline following the onset, and that this accelerated decline contributes to their poorer prognosis.

We also would make note of the test used to assess premorbid IQ. NART is a reading test suitable for adults, providing an estimate of general cognitive ability before the onset of a disorder affecting cognitive functions. Unlike other disorders and conditions for which the NART is often used, EOS develops and debuts at an age where cognitive abilities are not yet fully developed. The similarity in NART scores between the EOS and AOS groups indicates that the test can be used to assess premorbid IQ also in such conditions.

Longitudinal studies would be an optimal design for studying development of cognitive abilities. Such studies can elucidate cognitive trajectories, including possible cognitive decline in patients, and would provide stronger evidence about relationships between age of onset and cognition. Some earlier longitudinal studies have investigated various cognitive measures in adolescent patients with EOS. For instance, Frangou and colleagues compared cognitive functioning of 20 EOS patients, at baseline 15 and at follow-up 19 years old, with 20 HC. They found deterioration in verbal memory and attentional control in patients while HC showed increase in the same parameters (Frangou et al., 2008). Øie and colleagues studied 15 EOS patients, 19 patients with ADHD and 30 HC, aged 12–16 years at the baseline assessment (Oie et al., 2010). After 13 years, the EOS patients showed significant decline in verbal memory and arrest in development of attention and processing speed. The HC and the ADHD groups showed increases in these domains. The EOS group performed worse in the cognitive composite score, counted as the average of 9 cognitive measures, compared to the HC and the ADHD group at baseline, and at follow-ups after 13 and 25 years (Oie et al., 2020). Finally, Jepsen's study on cognition in 17 EOS, 11 patients with other early onset, non-organic, non-affective psychoses and 38 HC with a mean age of 15 years at baseline, showed increases in all studied cognitive measures in both HC and patients. Interestingly, the developmental progression of attention and set shifting was significantly worse in EOS patients than in HC at 5-year follow-up (Jepsen et al., 2010). Such longitudinal studies often have low numbers of participants, and most have relatively short duration of follow-up. A cross-sectional design which allows for the inclusion of more patients of different age and duration of schizophrenia overcomes some of these limitations.

The present study has some strengths and limitations that warrant mentioning. A strength of the study is that we have studied a well-characterized sample of patients and HC where several potential confounding factors were accounted for. A limitation is that the suggested IQ decline in both EOS and AOS patients is based on estimated measures in adults and not longitudinal data. Additionally, the cross-sectional design prevents us from determining the specific timing of the IQ decline throughout the lifespan.

Our findings bear potential clinical implications. The observed lower IQ among patients with EOS offers a potential explanation for the prognostic severity and diminished functional outcome within this patient cohort. Moreover, the early onset may contribute to the heterogeneity within the broader population of patients with schizophrenia. In addition, EOS patients usually experience a longer period of untreated psychosis, as our results also indicate, which may coincide with critical periods of cognitive, social, and academic development (Stentebjerg-Olesen et al., 2016). Antipsychotic medication effectively addresses positive symptoms but demonstrates limited, if any, efficacy against cognitive deficits and functional impairments (Harvey et al., 2016). Considering the above, it is important to implement early identification of cases to allow for psychosocial intervention strategies such as cognitive remediation. Such interventions have been shown to be efficacious for adults (Vita et al., 2021) as well as adolescents with schizophrenia (Puig et al., 2014; Ueland and Rund, 2005) and could improve long-term outcomes.

To conclude, we found that adults with an early-onset of schizophrenia had lower current full-scale, performance and verbal IQ than adult-onset patients. The patient groups showed similar premorbid IQ levels, and our study suggests that patients with EOS experience a steeper decline in cognitive abilities compared to AOS. Further research is needed to investigate underlying neurodevelopmental processes contributing to this decline. The current findings highlight the significance of early detection and interventions focused on preserving cognitive functions.

## CRediT authorship contribution statement

**Tereza Calkova:** Visualization, Project administration, Methodology, Formal analysis, Conceptualization, Writing – review & editing, Writing – original draft. **Anja Vaskinn:** Supervision, Project administration, Methodology, Investigation, Conceptualization, Writing – review & editing. **Lynn Mørch-Johnsen:** Methodology, Conceptualization, Writing – review & editing. **Runar Elle Smelror:** Methodology, Conceptualization, Writing – review & editing. **Kjetil Nordbø Jørgensen:** Methodology, Investigation, Conceptualization, Writing – review & editing. **Laura A. Wortinger:** Methodology, Conceptualization, Writing – review & editing. **Simon Cervenka:** Supervision, Methodology, Writing – review & editing. **Karin Collste:** Supervision, Methodology, Writing – review & editing. **Beathe Haatveit:** Investigation, Data curation, Writing – review & editing. **Christine Mohn:** Investigation, Data curation, Writing – review & editing. **Anne Margrethe Myhre:** Methodology, Conceptualization, Writing – review & editing. **Erik G. Jönsson:** Supervision, Methodology, Writing – review & editing. **Nils Eiel Steen:** Project administration, Methodology, Investigation, Writing – review & editing. **Ole A. Andreassen:** Resources, Project administration, Methodology, Funding acquisition, Data curation, Writing – review & editing. **Ingrid Melle:** Project administration, Methodology, Writing – review & editing. **Ingrid Agartz:** Supervision, Project administration, Methodology, Funding acquisition, Conceptualization, Writing – review & editing. **Torill Ueland:** Project administration, Methodology, Investigation, Conceptualization, Writing – review & editing. **Dimitrios Andreou:** Supervision, Project administration, Methodology, Formal analysis, Conceptualization, Writing – review & editing.

## Ethical standards

The authors assert that all procedures contributing to this work comply with the ethical standards of the relevant national and institutional committee on human experimentation and with the Helsinki Declaration of 1975, as revised in 2008. The study was approved by the Regional Committee for Medical Research Ethics (REC South East Norway). Written informed consent was obtained from all participants.

## Funding

This work was supported by the 10.13039/501100006095South-Eastern Norway Regional Health Authority (2019-108) and the 10.13039/501100005416Research Council of Norway (223273).

## Declaration of competing interest

OAA is a consultant to HealthLytix, and received speaker's honoraria from Lundbeck and Sunovion. IA received speaker's honoraria from Lundbeck. All other authors reported no potential conflicts of interest.

## Appendix A Supplementary data

Supplementary material Image 1
