# Supplementary material for: Early versus adult onset of schizophrenia: an examination of premorbid and current IQ
Source: Schizophr Res Cogn. 2025 Oct 14;43:100397. doi: 10.1016/j.scog.2025.100397 (PMC12549396; doi:10.1016/j.scog.2025.100397)
Supplement: Supplementary file 1 — Supplementary material [file mmc1.docx]

**Supplementary material**

**ANCOVA assumptions for the main models on FIQ, PIQ and VIQ (early-onset vs. adult-onset of schizophrenia)**

In the ANCOVA on FIQ, there was homogeneity of variances evaluated with Levene’s test, p=0.736. The residuals of the overall model were approximately normally distributed determined by visual inspection. There were no outliers (studentized residuals greater than three standard deviations). In the ANCOVA on PIQ, there was homogeneity of variances evaluated with Levene’s test, p=0.676. The residuals of the overall model were approximately normally distributed determined by visual inspection. There were no outliers. In the ANCOVA on VIQ, there was homogeneity of variances evaluated with Levene’s test, p=0.533. The residuals of the overall model were approximately normally distributed determined by visual inspection. There were no outliers.

**Analysis restricted to schizophrenia**

To rule out that the IQ differences between EOS and AOS were driven by differences in diagnostic distribution, we conducted an additional ANOVA on full-scale IQ restricted to patients with schizophrenia (72 EOS and 214 AOS). EOS patients still had significantly lower IQ than AOS patients, F(1,284) = 5.07, p = 0.025.

**Total variance explained by illness-related variables**

We conducted three regression analyses to assess the variance explained collectively by illness-related variables (AUDIT, DUDIT, duration of illness, duration of untreated psychosis, PANSS, antipsychotic use, and CPZ dosage) in relation to the IQ measures. For FIQ, PIQ, and VIQ, these illness variables together accounted for 2.7%, 2.5%, and 5.4% of the variance (adjusted R²), respectively. Collinearity diagnostics indicated that multicollinearity was not present, confirming that the predictors did not exhibit considerable overlap.

**Supplementary Tables**

|  | EOS (n=99) | AOS (n=282) |
| --- | --- | --- |
| Schizophrenia (%) | 72.7 | 75.9 |
| Schizoaffective disorder (%) | 24.2 | 12.8 |
| Schizophreniform disorder (%) | 3 | 11.3 |

**Suppl. Table 1.** Diagnostic breakdown in the early-onset schizophrenia (EOS) vs. adult-onset schizophrenia (AOS) groups
